# Early monitoring of inlay wear after total knee arthroplasty on plain radiographs using model-based wear measurement

**DOI:** 10.1038/s41598-024-68383-x

**Published:** 2024-08-06

**Authors:** Crystal Kayaro Emonde, Christof Hurschler, André Breuer, Max-Enno Eggers, Marcel Wichmann, Max Ettinger, Berend Denkena

**Affiliations:** 1 Laboratory for Biomechanics and Biomaterials, Hannover Medical School, Department of Orthopaedic Surgery, DIAKOVERE Annastift, Anna Von Borries Str. 1-7, 30625 Hannover, Germany; 2 Institute of Production Engineering and Machine Tools, Leibniz University Hannover, An Der Universität 2, Garbsen, 30823 Hannover, Germany; 3 Department of Orthopaedic Surgery, Pius-Hospital Oldenburg, University Clinic for Orthopaedics and Trauma Surgery, Georgstraße 12, 26121 Oldenburg, Germany

**Keywords:** Inlay wear, In-vivo wear, Standard radiograph, Model-based, Minimum thickness, Polyethylene wear, Medical research, Biomedical engineering, Implants

## Abstract

Wear of the ultra-high molecular-weight polyethylene (UHMWPE) component in total knee arthroplasty contributes to implant failure. It is often detected late, when patients experience pain or instability. Early monitoring could enable timely intervention, preventing implant failure and joint degeneration. This study investigates the accuracy and precision (repeatability) of model-based wear measurement (MBWM), a novel technique that can estimate inlay thickness and wear radiographically. Six inlays were milled from non-crosslinked UHMWPE and imaged via X-ray in anteroposterior view at flexion angles 0°, 30°, and 60° on a phantom knee model. MBWM measurements were compared with reference values from a coordinate measurement machine. Three inlays were subjected to accelerated wear generation and similarly evaluated. MBWM estimated inlay thickness with medial and lateral accuracies of 0.13 ± 0.09 and 0.14 ± 0.09 mm, respectively, and linear wear with an accuracy of 0.07 ± 0.06 mm. Thickness measurements revealed significant lateral differences at 0° and 30° (0.22 ± 0.08 mm vs. 0.06 ± 0.06 mm, respectively; t-test, p = 0.0002). Precision was high, with average medial and lateral differences of − 0.01 ± 0.04 mm between double experiments. MBWM using plain radiographs presents a practical and promising approach for the clinical detection of implant wear.

## Introduction

Total knee arthroplasty (TKA) is an end-stage surgical procedure that is performed on patients suffering from degenerative knee joint disease, and has a reported 10-year survival rate of over 90%^1,2^. Nevertheless, implant failure due to the wear of the polymeric bearing component remains a major cause of implant revision, especially in patients younger than 55 years old^2^. Ultra-high molecular weight polyethylene (UHMWPE) is the gold standard material for the bearing component commonly referred to as the inlay or insert, which plays a critical role in facilitating the low-friction articulation of the TKA components in a manner similar to the cartilage^3^.

The wear of the inlay is a potential cause of TKA failure that is associated with pain, instability, and malfunctioning of the TKA^4^. It is influenced by the gender, Body Mass Index (BMI), and weight of the patient as well as the surgical technique used, design of the implant, and pre-existing diagnoses and illnesses (e.g., osteoarthritis, rheumatoid arthritis, or poliomyelitis)^5–7^. Wear-generated inlay debris has been reported to trigger osteolysis and bone resorption that eventually lead to the loosening of the joint replacement^8,9^. According to the annual report by the German Arthroplasty Registry, inlays accounted for 20% of revised components in TKA revisions in 2022^10^.

The number of TKA procedures in Germany is expected to increase by 45% by 2040 (compared with those in 2016), and more revisions are consequently expected^11,12^. Revisions are often performed late, once severe wear of the inlay has occurred. The instability of a severely worn implant can lead to a significant loss of the remaining intrinsic stability of the knee joint^7^. Accurate non-invasive methods that allow for the early monitoring and in-vivo detection of the wear of the inlay can help mitigate complications that often arise in the course of wear-induced failure and revision of the implant.

The main challenge to the detection of polythene wear in TKA at present is that it often goes undetected unless it is sufficiently substantial to be radiographically visualized^13^. The relevant studies have thus relied on simulators or retrospective analyses of retrievals to assess inlay wear^14,15^. Conventional methods of assessing the progression of wear involve assessing the minimal distance between the femoral component and the tibial baseplate in a 2D plain radiograph by using manual or computer-assisted techniques^16,17^. This distance, clinically referred to as the minimum joint space width (mJSW), is equivalent to the thickness of the inlay, which appears radiolucent in an X-ray radiograph. These techniques have been reported to often be unreliable such that they yield large errors (> 1 mm)^18^. Furthermore, an anterior or posterior tilt of the tibial baseplate can lead to the inaccurate estimation of the thickness of the inlay^6,17^.

Hoshino et al. introduced 2D-to-3D matching to estimate the wear of the inlay in-vivo. They took standard anteroposterior radiographs of patients with implants, with the knee joint encased in a plastic calibration cylinder that had been embedded with tantalum balls, the positions of which defined a local coordinate system. The 3D pose of the implant could be extracted from the radiograph and data on the numerical geometry of the components of the implant^6^. Their results revealed that wear and creep of the inlay could be estimated to an accuracy of within 0.1 mm.

Van Ijsseldijk et al. proposed a model-based wear measurement (MBWM) approach to assess implant wear^19^. This technique is based on model-based Roentgen stereophotogrammetric analysis (MBRSA), which has been successfully used to study implant migration^19^. Using MBRSA software, MBWM performs 2D-to-3D registration of the 3D surface models of implant components to their silhouettes on a 2D plain radiograph. Unlike conventional MBRSA analysis, which requires specialized stereo-images and a physical calibration box, MBWM only requires a digital X-ray image and a virtual calibration box containing radiograph information i.e. the film-focus distance and image resolution^13^. After registration, the 3D poses of implant components are acquired, and the mJSW is determined by assessing the minimum distance between the surface models relative to each other^18^. A change in mJSW over time represents linear wear.

In their first in-vitro investigation of this technique, van Ijsseldijk et al. found that MBWM estimated the thickness of a PMMA plate with an accuracy of 0.14–0.15 mm and a reproducibility of 0.20 mm^18^. The accuracy was lower than that obtained from MBRSA because the calibration box was absent and only a single X-ray source was used^20^. Despite this, MBWM exhibited higher accuracy than the conventional clinical methods of wear assessment^18,19^. MBWM provides a practical solution for clinical applications, as it utilizes readily available routine follow-up radiographs eliminating the need for additional X-ray exposure and costly calibration equipment.

To the best of our knowledge, few studies in the area have investigated model-based wear measurement particularly using standard radiographs. In previous studies, PERSPEX^®^/PMMA blocks have been used as specimens in in-vitro experiments^13,20^. In our study, we milled UHMWPE standard inlays, similar to those used clinically to replicate the empirical situation.

Our primary objective was to investigate the accuracy and precision of MBWM in estimating the minimum thickness of these standard inlays in an in-vitro setting. Secondarily we evaluated MBWM’s accuracy in estimating the linear wear after subjecting the inlays to accelerated wear tests in a testbed. The inlays were radiographically imaged on a phantom knee model at flexion angles ranging between 0° and 60° before and after wear generation. After 2D-to-3D registration, measurements were taken for both medial and lateral condyles of the standard inlays. The results were compared with reference measurements taken with a coordinate measurement machine (CMM). The findings of this study could pave the way for a highly accurate technique for early clinical assessment of implant wear, enabling timely detection and intervention. As this technique only requires clinical radiographs and MBRSA software, it is both practical and affordable.

## Materials and methods

### Standard inlays

**Figure 1:**
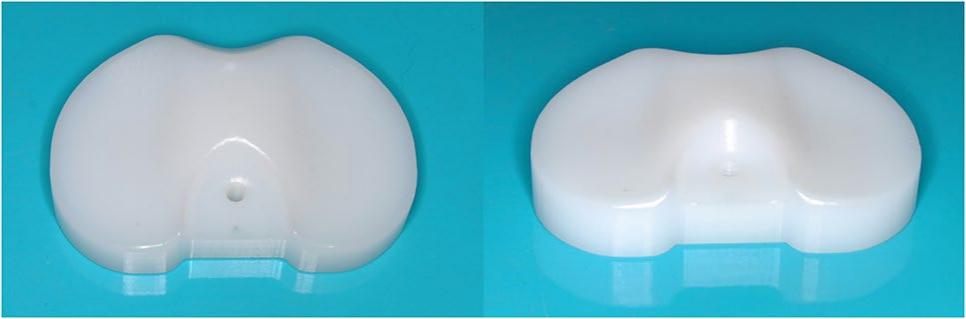
Two views of a standard inlay milled from UHMWPE 1000.

Standardized inlays were used to investigate the accuracy of wear detection under reproducible conditions involving different degrees of wear at different locations. We milled six inlays with mediolateral and anteroposterior measurements of 76 mm and 50 mm, respectively, and thicknesses of 10 mm, 14 mm, and 16 mm from non-crosslinked UHMWPE 1000 in our research workshop (Fig. 1). The articulating surfaces of the inlays were designed to match the radius of a size 6 Stryker Triathlon cruciate-retaining (CR) femur component, and to provide full congruency with the femur component during wear generation and imaging in the phantom model. To simplify the geometry of the inlay, and enable its easy insertion and removal from the testbed and the phantom model, its backside was designed to be flat. Hence, the inlays could not be snap-fitted into the tibia component. A screw hole was incorporated on the inlay to enable its secure attachment to the tibial tray of the testbed during wear generation.

### Counter-surface roughening

Prior to wear generation, the surface of a size 6 Stryker Triathlon CR femur (Stryker European Operations B.V., Amsterdam, Netherlands) was roughened in a sandblasting machine (Powerplus Tools, Powerplus Tools GmbH, Ahlhorn, Germany). Light scratches were observed microscopically on the femur surface prior to the roughening process. With a reported linear rate of wear of 0.1–0.2 mm/year, the UHMWPE inlays would require millions of cycles and months of testing to achieve significant wear^21–23^. Sandblasting was carried out to accelerate wear generation and yield a substantial amount of wear. Desjardins et al. reported a 5.6-fold increase in the rate of wear of the UHMWPE articulated against a cobalt–chromium (CoCr) femur component that had been roughened to 0.172 µm^24^. The abrasive powder consisted of aluminum oxide (Al_2_O_3_) powders (Edelkorund F40) with a particle size of 355–500 µm. Sandblasting on each condyle was conducted at an air pressure of 5 bar for a duration of 30 s at a constant distance of 30 cm. The component was ultrasonically cleaned after roughening.

A laser profilometer (μScan, NanoFocus AG, Buschhausen, Germany) was used to characterize the roughness of the specimens. The femur was mounted on a steel holder, and measurements taken at four locations with dimensions of 10 × 10 cm on the anterior and posterior regions of each condyle. The values of surface roughness of all points were averaged and quantified by using the average roughness (R_a_) and the maximum peak height (R_p_). MountainsMap software (Version 8, Digital Surf, Besançon, France, https://www.digitalsurf.com/) was used for surface analysis.

### Wear generation

The wear patterns were generated in an in-house testbed that was designed to perform simplified simulations of knee wear. Three standard inlays with the thicknesses mentioned above were subjected to wear testing, while the other three remained unworn. The parameters of wear were set based on the limits of the testbed. The inlays were secured to the tibial tray of the testbed by using screws, and were subjected to 14,500–28,800 cycles of wear at a frequency of 1 Hz and flexion angles ranging from 0° to 28° to generate diverse wear patterns. Lubrication was provided by using 40 ml of an undiluted 0.9% NaCl solution (Fresenius Kabi GmbH, Bad Homburg, Germany). A compressive load of 125–234 N was applied during testing, and the ambient temperature was maintained at 20–21 °C throughout the test. The femur component was ultrasonically cleaned before and after each test to remove debris and the transfer film from its surface.

### Phantom model/X-ray acquisition

**Figure 2:**
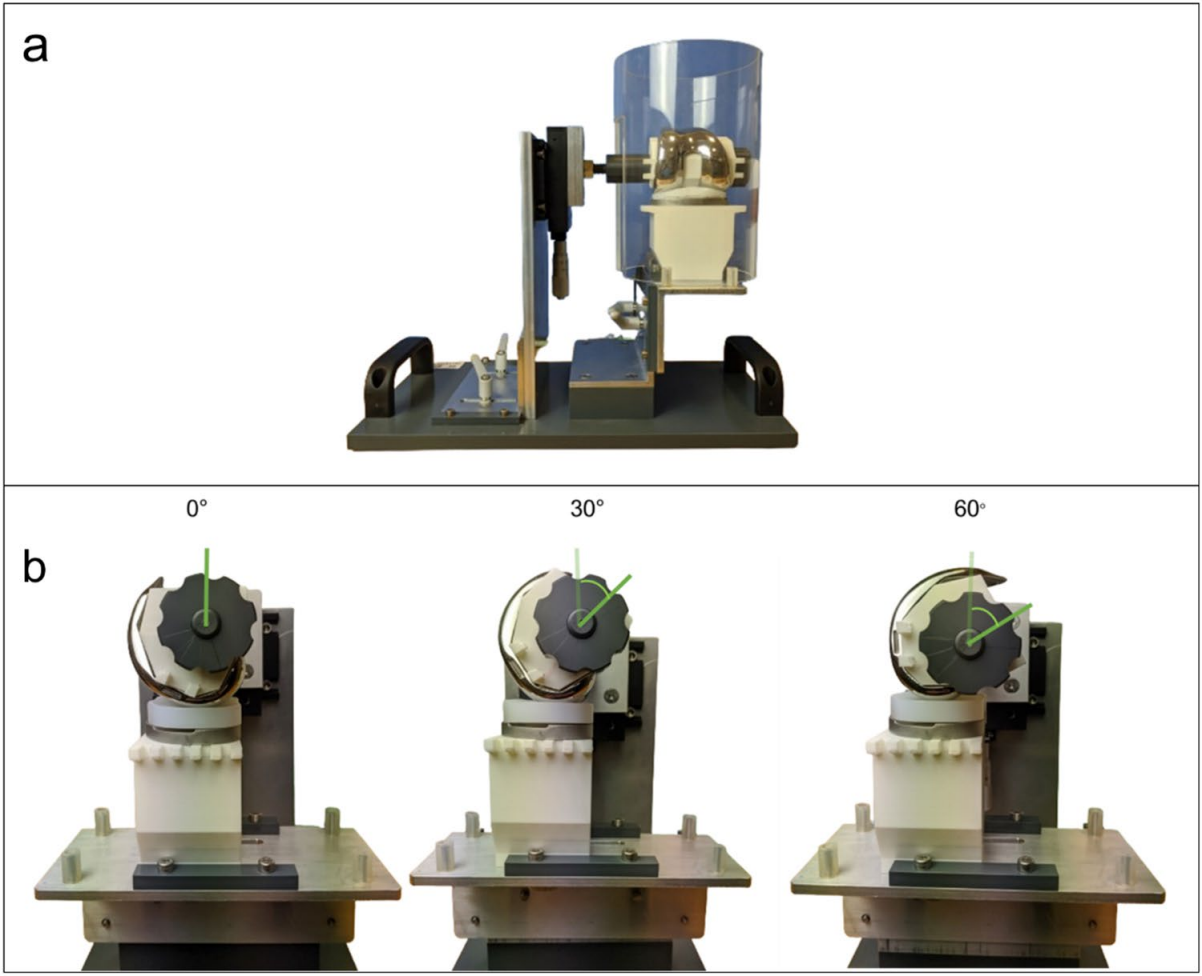
(**a**) Phantom knee model fitted with TKA components, and a PMMA tube to represent the soft tissue. (**b**) Lateral view of phantom knee model with femur component flexed at angles of 0°, 30°, and 60°.

A phantom knee model was used to position the TKA components in views similar to those used in clinical knee imaging. The phantom model was equipped with a translational micromanipulator and adjustment levers to enable the simulation of physiological movements about three axes, i.e., anterior–posterior, medio–lateral, and superior–inferior translation, as well as the flexion of the femur component about its rotational center (Fig. 2). The Stryker Triathlon CR fixed-bearing (size 6, right) components were fitted into 3D-printed polymer components that represented the bone. A 5-mm-thick cylindrical tube composed of Plexiglas was placed around the implant components during imaging to mimic the attenuation of X-rays by the soft tissue.

Each standard inlay was introduced between the components of the implant on the phantom and the adjustment levers used to ensure optimum conformity and positioning. As the bottom of the milled inlay was devoid of the locking features that are available in the commercial variant, it could not be snap-fitted into the tibial baseplate. Instead, the inlay, which was marginally larger than the tibial baseplate, was set onto the peripheral rim of the tibial component, and the height of the peripheral rim was subtracted from the mJSW (Fig. 2). This also allowed for easy repeated insertion and removal of the inlays. Silicone bands were used to ensure contact and prevent condyle lift-off. For each inlay, AP radiographs were acquired at flexion angles of 0°, 30°, and 60° by using a ceiling-mounted digital X-ray system (DigitalDiagnost, Philips Medical Systems, Amsterdam, Netherlands), with imaging parameters of 1.7 mAs and 63 kV, and a source-to-detector distance (SDD) of 1.15 m. In total, 18 radiographs, were captured at a resolution of 96 dots per inch (DPI).

### Analysis of inlay thickness and wear

The fluoroscopy module of the MBRSA software (Model-based RSA, version 4.10, RSAcore, Leiden, The Netherlands, https://rsacore.nl/model-based-rsa/) was used to locate the femoral and tibial components relative to each other. This method deviates from the traditional dual-plane MBRSA in that only a single radiograph/plane is used instead of the typical stereo pair. A 2D-to-3D technique of registration is used in this mode of operation. No physical calibration box is required; instead, a specialized virtual flat-panel calibration box (Leiden Single Flat Panel Fluo, Leiden, Netherlands) containing image-related information (i.e., SDD and DPI) is loaded into the software. It does, however, require that the distances between the X-ray source, the knee, and the plane of the sensor or film are known, and that the image plane is normal to the X-ray source. X-ray files in DICOM format and 3D surface models of the components of the implant were used for the analysis.

Reverse-engineered 3D surface models of the components of the implant were generated by using a structured light scanner (HP 3D SLS Pro S3, HP HQ-TRE, Boeblingen, Germany) with an accuracy of 0.05 mm. 3D scanner software (HP 3D Scan Software, version Pro v5, https://support.hp.com/ch-de/product/setup-user-guides/hp-3d-scan-software/model/14627413) was used to register the scan data and create stereolithography (STL) models. The final number of polygons of the STL models was reduced to 5,000 using 3D modelling software (Blender, version 3.4.1, https://www.blender.org/) to reduce the computation time. The original scans and their reduced models were registered in 3D mesh processingsoftware (CloudCompare, version 2.13.0, https://www.cloudcompare.org/) and deviations of − 0.4–1.0 mm and − 0.4–0.3 mm were obtained for the femur and tibia respectively.

The contours of the components of the implant in the radiograph were detected in the software by using Canny edge detection algorithms. The contours of the surface model were also detected and projected to the radiograph. The two sets of contours were matched, and the differences in pose estimation between them were minimized by using the DIF DHSAnn algorithm provided in the software^25^. A 3D reconstruction of the pose (position and orientation) of the components of the implant was thus obtained.

Subsequent calculations of the minimum thickness of the inlay and its linear wear were conducted by using a closest-points algorithm. Conventionally, the minimum thickness usually means the distance between the femur and the top of the tibial baseplate. In this study, we measured the minimum distance from the femur to the plane of the peripheral rim, where the inlay rested, because direct inlay–baseplate contact was absent. Linear wear was regarded as the change in this separation before and after wear generation. Measurements were determined for both the medial and the lateral condyles.

### Direct measurement of wear

To assess the accuracy of this method in terms of estimating both the minimum thickness of the inlay and its linear wear, we compared the model-based estimates with reference values taken by using a tactile CMM (Leitz Reference XI, Probe: HP-S-X5 HD, Hexagon, Stockholm, Sweden) with an accuracy of < 0.1 μm.

**Figure 3:**
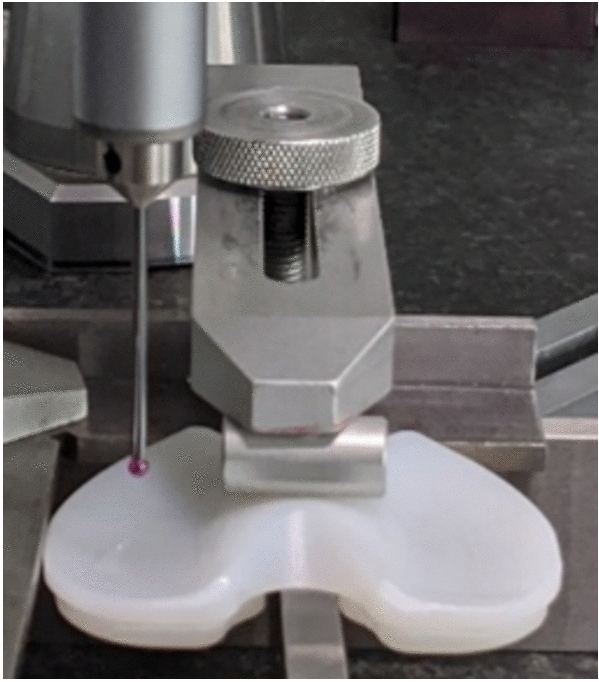
Setup of the CMM, with the ball probe above the condyle of the inlay.

Each inlay was securely clamped in place (Fig. 3) and evaluated for horizontal alignment/straightness. A ball probe was used to determine the minimum thickness from the top of each condyle over an area of 2 × 10 mm to the bottom of the inlay. To guarantee measurement at the deepest point on the respective condyle, the straightness at the relevant point was assessed, and the measurement was recorded when the straightness of the inlay was within the acceptable level of tolerance. The ambient temperature was maintained at 20 °C during the entire measurement.

### Statistical analysis

The accuracy of the measurements was determined by calculating the mean absolute error between the model-based measurements and those of the CMM. Precision was evaluated by using the repeatability of double experiments of the same radiographs, and Bland–Altman plots with a 95% limit of agreement (LoA) were used to assess the agreement between the measurements. The measurement error was used to assess bias. Paired t-tests were conducted to determine the significance of differences in measurements of the mJSW obtained at different poses, with the significance set to p < 0.05. Moreover, the one-way analysis of variance (ANOVA) was applied to assess the differences in measurements of linear wear at all flexion angles.

The following equations were used for the calculations:

1 $$\text{Measurement error}=\text{ MBWM measurement}-\text{CMM measurement}$$

2 $$\text{Mean absolute error }\left(\text{MAE}\right)= \frac{1}{\text{n}}\sum_{\text{i}=1}^{\text{n}}|\text{MBWM}-\text{CMM measurement}|$$

3 $$\text{Linear wear}={\text{mjsw}}_{\text{new}}-{\text{mjsw}}_{\text{worn}}$$

## Results

### Roughening and wear generation

After roughening, the surface of the femur component gained a noticeably matte and porous appearance, and was rougher to the touch. The average roughness (R_a_) increased approximately tenfold from 0.08 to 0.86 µm, while the maximum peak height (R_p_) tripled from an initial value of 0.68–2.13 µm (Table 1). The 2D surface topography and roughness profiles of the femur prior to and after roughening, as well as the 3D surface profiles of the standard inlays after wear generation, were evaluated (Supplementary Figs. S1–S3 online).

**Table 1:** Parameters of roughness of the femur component before and after roughening treatment.

Femur component	R_a_ (µm)	R_p_ (µm)
Before roughening	0.08	0.68
After roughening	0.86	2.13

### Accuracy

**Figure 4:**
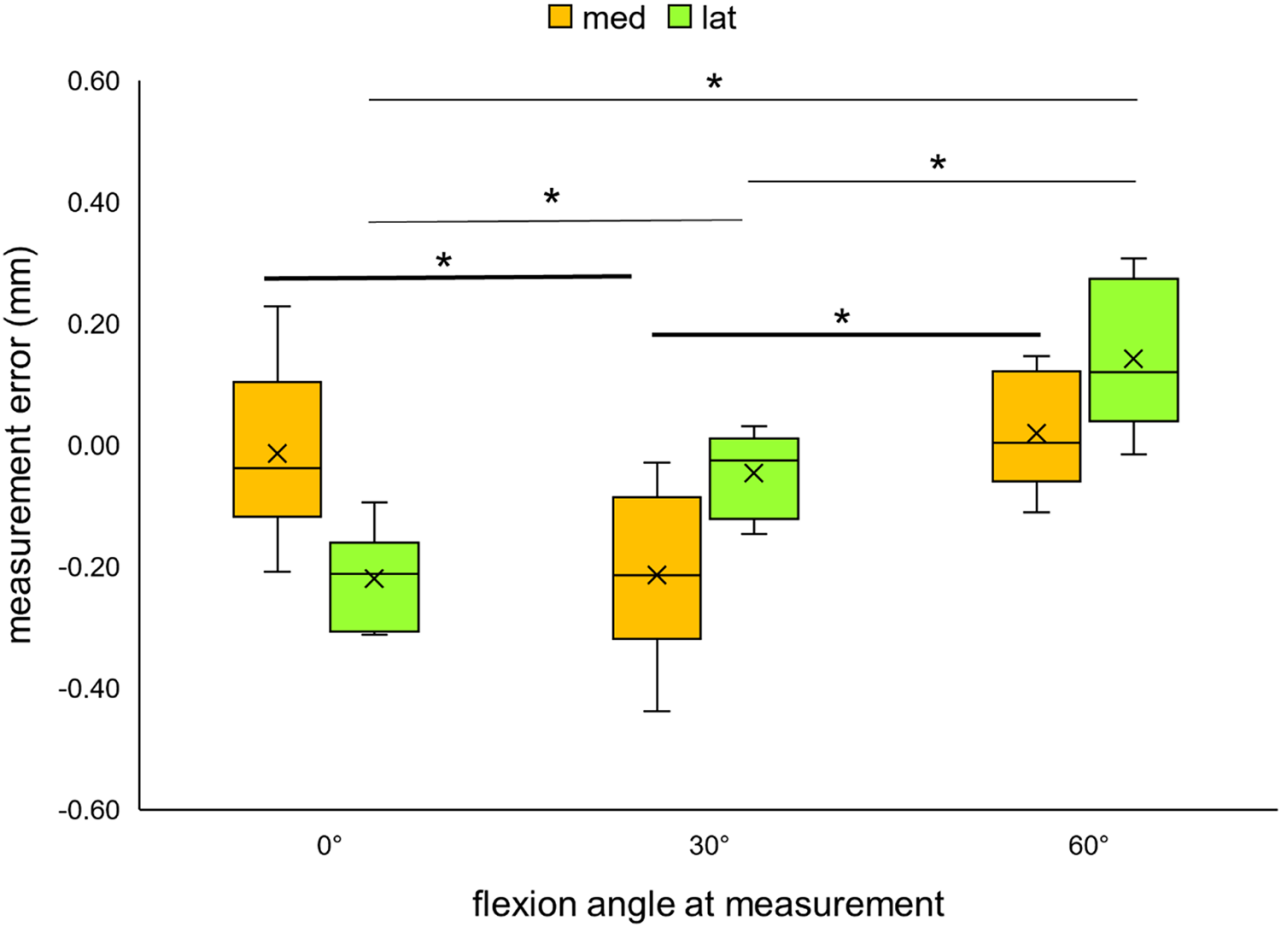
Box plots showing errors in the measurements of the minimum joint space width (mJSW) between the model-based and CMM measurements at the three flexion angles. The bold bars represent measurements of the medial condyle, while the bars in regular font represent those of the lateral condyle. The asterisk indicates a statistical significance of p < 0.05.

We evaluated the measurement error and accuracy of the model-based method in terms of estimating the mJSW of each condyle at the three flexion angles (Fig. 4, Table 2). A negative systemic bias was evident at flexion angles of 0° and 30° for both condyles, indicating that the model-based method had underestimated the mJSW at these flexion angles (− 0.01 ± 0.15 and 0.22 ± 0.08 mm, and − 0.21 ± 0.14 and − 0.05 ± 0.07 mm, medially and laterally, at 0° and 30°, respectively).

**Table 2:** Measurement error and mean absolute error (MAE) between the model-based and CMM measurements of the mJSW.

Flexion angle (°)	Condyle	Measurement error (mm)	SD	MAE (mm)	SD	MAE ± SD (both condyles)
0	med	− 0.01	0.15	0.11	0.09	0.17 ± 0.08
	lat	− 0.22	0.08	**0.22**	0.08	
30	med	− 0.21	0.14	0.21	0.14	0.22 ± 0.11
	lat	− 0.05	0.07	**0.06**	0.06	
60	med	0.02	0.10	0.08	0.05	0.14 ± 0.10
	lat	0.14	0.12	0.15	0.12	

Bold values are statistically significant *p* < 0.05, paired t-test.

The mean absolute results of the model-based method ranged from 0.06 to 0.22 mm, and its accuracy varied depending on the condyle and the flexion angle (0°, 30°, 60°) (Table 2). Average accuracies of 0.13 ± 0.09 mm and 0.14 ± 0.09 mm were obtained for measurements of the medial side and the lateral side, respectively, across all flexion angles. A statistically significant difference in accuracy was evident between the results for the lateral side at flexion angles of 0° and 30° (0.22 ± 0.08 mm vs. 0.06 ± 0.06 mm at 0° and 30°, respectively; t-test p < 0.05) (Table 2).

The average accuracy per condyle did not exhibit a large variation over the range of flexion angles, indicating that the mJSW could be estimated accurately within a range from 0.14 ± 0.10 to 0.22 ± 0.11 mm between 0° and 60° of flexion (Table 2).

Linear wear was defined as the difference in the mJSW between inlays before and after wear tests. The accuracy of the measured linear wear (MAE) ranged from 0.05 ± 0.04 to 0.09 ± 0.09 mm across the three flexion angles (Table 3). A negative bias was evident at a flexion of 0°, suggesting that the model-based method had underestimated linear wear. Conversely, a positive bias was observed for the other two flexion angles, indicating an overestimation of linear wear by the model-based method (Table 3). Nevertheless, this overestimation was < 0.05 mm for all flexion angles, indicating that no large variations were evident in measurements of linear wear. The results of ANOVA showed no statistically significant difference in the accuracy of measurements of linear wear by the model-based method among the three flexion angles.

**Table 3:** Mean absolute error (accuracy) of measurements of linear wear at the three flexion angles. Each group of flexion angles represents six measurements.

Flexion angle (°)	Mean measurement error ± SD	MAE of linear wear ± SD
0	− 0.02 ± 0.10	0.07 ± 0.06
30	0.00 ± 0.06	0.05 ± 0.04
60	0.03 ± 0.12	0.09 ± 0.09

### Precision

**Figure 5:**
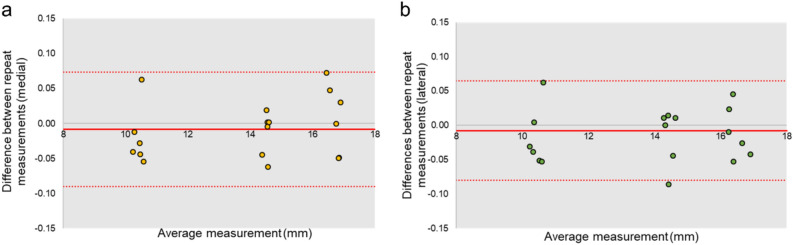
Bland–Altman plots showing the agreement between repeated measurements of the mJSW for the (**a**) medial and (**b**) lateral condyles for 18 measurements. The horizontal line represents the average difference and the dashed lines show the limits of agreement.

To assess the precision of the model-based method, a single researcher performed double measurements of the mJSW across 18 radiographs encompassing six inlays at the three flexion angles considered in this study. Eighteen pairs of measurements were compared for each condyle (Fig. 5). The measurements predominantly fell within the limits of agreement (− 0.09 and − 0.08 mm medially and laterally, respectively). Average differences between repeated measurements showed a minor deviation of − 0.009 ± 0.042 mm on the medial side and that of − 0.008 ± 0.037 mm on the lateral side, indicating good agreement between the first and second measurements.

## Discussion

Wear of the polymer inlay remains a challenge that compromises the long-term success of TKA. Innovative methods are needed to enable early monitoring that can enable early diagnosis and timely intervention before implant failure and the progression of joint degeneration.

In this study, we investigated the accuracy and precision of a model-based technique of wear measurement based on MBRSA in an in-vitro setting. We investigated how accurately this method could estimate the mJSW of standard milled inlays with known thicknesses against reference values. We also investigated whether this method could detect submillimeter-level wear in the inlays, and examined its repeatability as well as the effect of the pose (flexion) of the implant on measurements of the mJSW and linear wear. Through these evaluations, we aimed to provide insights into the reliability of the model-based approach for the assessment of wear in orthopedic applications.

The main advantage of the above method is that it foregoes the use of additional specialized equipment that is required by conventional MBRSA. Fontalis et al. have encouraged the use of either standard clinical radiographs or a modified setup in MBRSA research to compensate for the unavailability of specialized equipment^26^. Van Ijsseldijk et al. proposed the integration of a device in the in-vitro setup to replicate the attenuation of the soft tissue in X-rays that is prevalent in clinical images^18^. In this study, we incorporated a PMMA tube that encased the components during imaging. A similar tube was used by Seehaus et al. to represent the soft tissue surrounding the hip^27^. We found that the contours were easily detectable in the image even with X-ray attenuation. In clinical contexts, this may be a little more challenging in situ due to the effects of the bone or tissue attenuation.

We obtained mean absolute errors (accuracy) of the mJSW measurements ranging from 0.06 ± 0.06 mm to 0.22 ± 0.08 mm, with variations across condyles and flexion angles. Collectively, MAEs of 0.13 ± 0.09 mm and 0.14 ± 0.09 mm were obtained for the medial side and the lateral side, respectively, across all flexion angles. These results are closely aligned with the findings of Van Ijsseldijk et al., who reported an accuracy of 0.14–0.15 mm laterally in fixed and mobile TKAs in standard radiographs^18^. Another study by the same group reported an accuracy of 0.1 mm when the effect of the flexion angles (0°, 30°, 45°) was considered^20^. However, it is important to note that this study used a conventional MBRSA setup, which typically yields a significantly higher theoretical accuracy than a 2D-to-3D registration on standard radiographs. Furthermore, both the above-mentioned studies used PMMA plates, with measurements taken exclusively on one condyle.

Although the design goal of our phantom was to obtain the highest possible accuracy, we found a statistically significant difference in the accuracy of measurements of one condyle, particularly between flexion angles of 0° and 30° for the lateral condyle. We hypothesize that this may have stemmed from potential condyle lift-off during flexion, or from variations in the force exerted by the silicone bands on the phantom.

Highly accurate measurements of linear wear were obtained, with MAE ranging from 0.05 ± 0.04 mm to − 0.09 ± 0.09 mm across all flexion angles. The MAE across all flexion angles was 0.07 ± 0.06 mm. The higher accuracy of linear measurements can be attributed to the larger sample size used. The mJSW measurements were aggregated irrespective of condyle, and yielded 18 measurements.

Nevertheless, a negative bias (mean error) was observed in the linear measurements at 0°, indicating that the model-based method might have underestimated the actual wear. This variation may have resulted from the difference in contact area between the femur component compared with that of the CMM’s ball stylus. Owing to its larger area of contact, the femur component might have come into contact with wear pools that might have been missed by the ball probe, which took measurements over a much smaller area and with a relatively small stylus (Ø = 3 mm diameter).Van Ijsseldijk found that the outcome of the measurement was dependent on the location of contact^20^.

Past studies have shown that different wear pools may exist on the surface of the inlay, thus it is recommended that wear measurements be carried out at multiple flexion angles^28,29^. In our study we found no statistically significant difference in the linear wear observed at the three flexion angles. However, significant differences were observed in measurements of the mJSW laterally between flexion angles of 0° and 30°.

The results of precision showed good repeatability, with average differences in the medial and lateral measurements of − 0.009 ± 0.042 mm and − 0.008 ± 0.037 mm, respectively, between repeated measurements.

The final differences between the contours of the actual implant models and those detected in the radiograph after registration in the MBRSA software ranged from 0.09 to 0.12 mm. Ideally, this difference should be lower, and is dependent on the accuracy of the surface models used^30^. Variations in scan accuracy can stem from post-scan processing procedures, such as polygon reduction and smoothing that can lead to the loss of detail, and overlap between the original and reduced models^30^. We found deviations of − 0.4–1.0 mm and − 0.4–0.3 mm (femur and tibia respectively) between the original scans, consisting of ~ 2 million polygons, and the reduced scans consisting of 5,000 polygons. Values of final pose estimation below 0.05 mm can improve the pose estimation and enhance the accuracy of this technique.

Our study had the following limitations: due to the roughening of the femur surface and the use of a testbed with limited parameters, the patterns of wear did not fully replicate those observed clinically. Furthermore, the phantom model was not loaded physiologically, but with silicone bands, and this might have resulted in some variation in the points of contact, or in instability.

Nevertheless, model-based wear measurement by using standard radiographs is a practical and promising approach to the detection of TKA polyethylene wear that is accurate and precise. It is important to maintain consistency in the pose of the knee joint between follow-ups to enable accurate measurements of the mJSW and linear wear at consistent points of contact.

### Supplementary Information

Supplementary Figures.

## Data Availability

Data analyzed during the current study are available from the corresponding author upon reasonable request.
