# Supplementary material for: Early monitoring of inlay wear after total knee arthroplasty on plain radiographs using model-based wear measurement
Source: Sci Rep. 2024 Aug 6;14:18248. doi: 10.1038/s41598-024-68383-x (PMC11303532; doi:10.1038/s41598-024-68383-x)
Supplement: Supplementary file 1 — Supplementary Figures. [file 41598_2024_68383_MOESM1_ESM.pdf]

Supplementary: Early monitoring of inlay wear after total knee arthroplasty on plain radiographs using model-based wear measurement

Crystal Kayaro Emonde[1,*], Christof Hurschler[1], André Breuer[1], Max-Enno Eggers[2], Marcel Wichmann[2], Max Ettinger[3], Berend Denkena[2]

[1] Laboratory for Biomechanics and Biomaterials, Hannover Medical School, Department of Orthopaedic Surgery, DIAKOVERE Annastift, Anna von Borries Str. 1-7, 30625 Hannover, Germany

[2] Institute of Production Engineering and Machine Tools, Leibniz University Hannover, An der Universität 2, 30823 Garbsen, Hannover, Germany

[3] Department of Orthopaedic Surgery, Pius-Hospital Oldenburg, University Clinic for Orthopaedics and Trauma Surgery, Georgstraße 12 26121 Oldenburg, Germany

[*]Corresponding author: emonde.crystal@mh-hannover.de ORCID: 0000-0001-6557-3756

Figure S1. 2D surface topography images of the femur surface before roughening, along with the corresponding roughness profiles for the sampling lengths obtained from locations: (**a**) anterior left, (**b**) anterior right, (**c**) posterior left, and (**d**) posterior right using a laser profilometer. 2

Figure S2. 2D surface topography images of the femur surface after roughening, along with the corresponding roughness profiles for the sampling lengths taken from locations: (**a**) anterior left, (**b**) anterior right, (**c**) posterior left, and (**d**) posterior right using a laser profilometer. 3

Figure S3. 3D surface profile images of the worn regions on the left and right sides of the standard inlay surfaces, captured using a laser profilometer after wear testing in the testbed. 4

Figure S1

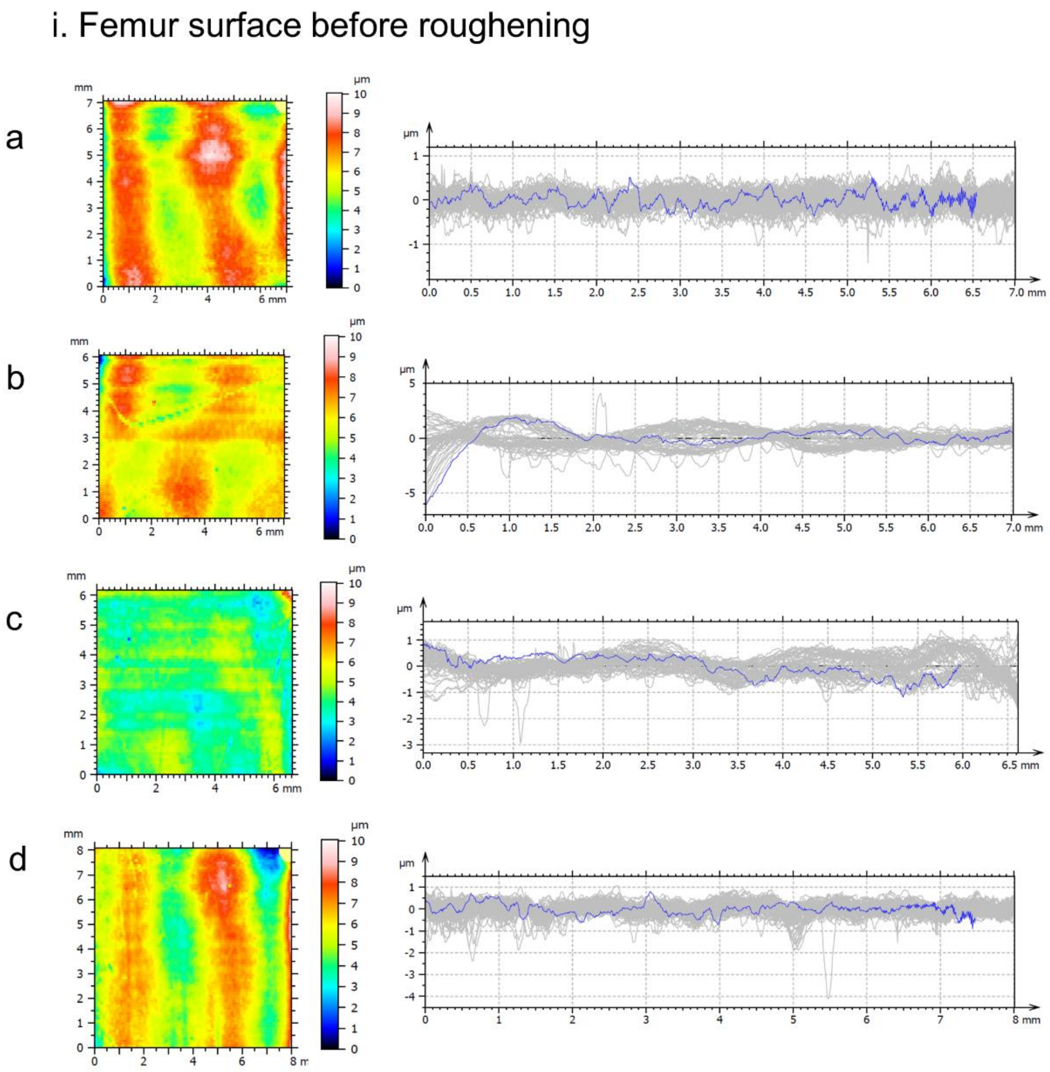

Figure S1. 2D surface topography images of the femur surface before roughening, along with the corresponding roughness profiles for the sampling lengths obtained from locations: (**a**) anterior left, (**b**) anterior right, (**c**) posterior left, and (**d**) posterior right using a laser profilometer.

Figure S2

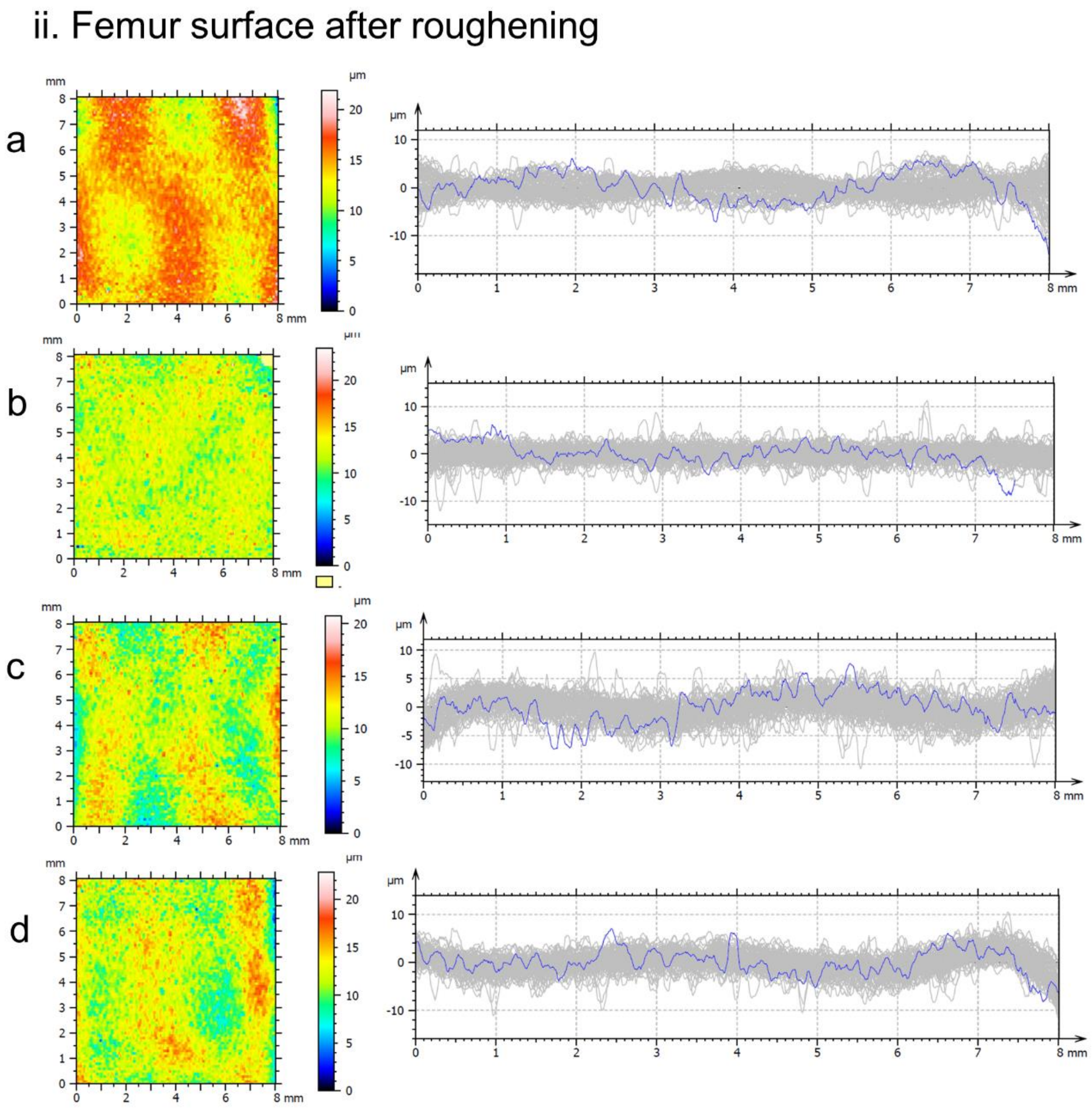

Figure S2. 2D surface topography images of the femur surface after roughening, along with the corresponding roughness profiles for the sampling lengths taken from locations: (**a**) anterior left, (**b**) anterior right, (**c**) posterior left, and (**d**) posterior right using a laser profilometer.

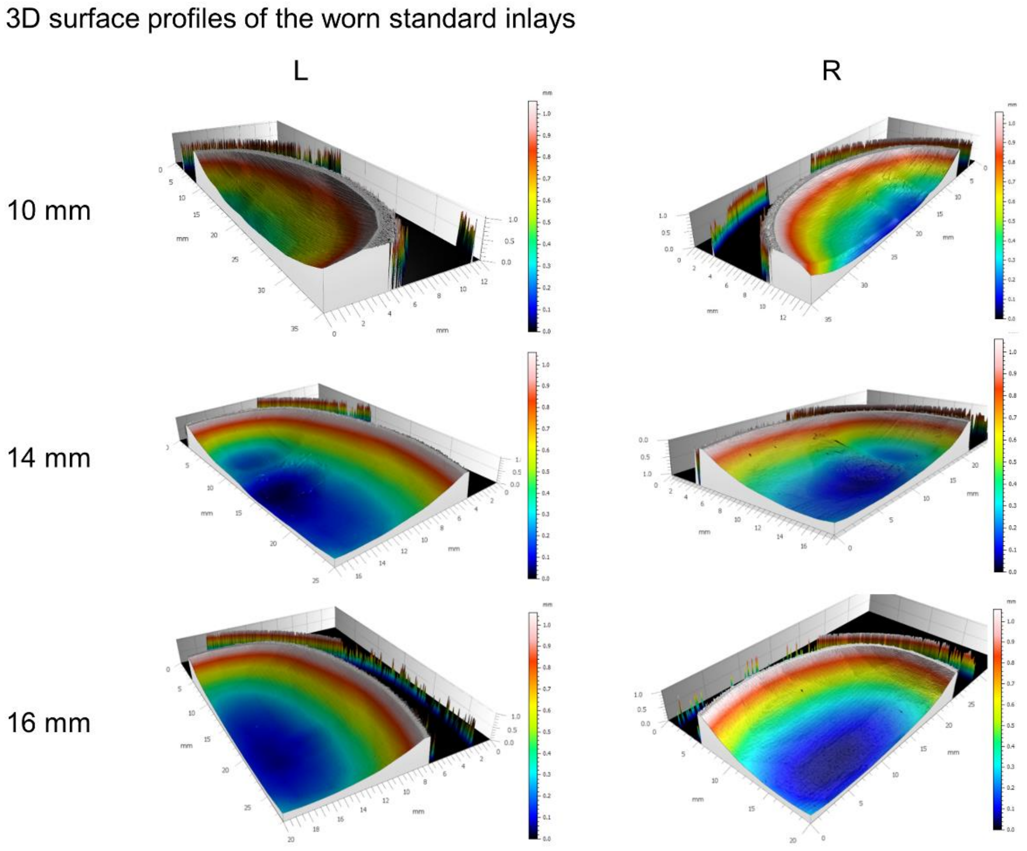

Figure S3. 3D surface profile images of the worn regions on the left and right sides of the standard inlay surfaces, captured using a laser profilometer after wear generation in the testbed.
